# SAR by Space: Enriching Hit Sets from the Chemical Space

**DOI:** 10.3390/molecules24173096

**Published:** 2019-08-26

**Authors:** Franca-Maria Klingler, Marcus Gastreich, Oleksandr O. Grygorenko, Olena Savych, Petro Borysko, Anastasia Griniukova, Kateryna E. Gubina, Christian Lemmen, Yurii S. Moroz

**Affiliations:** 1 BioSolveIT GmbH, An der Ziegelei 79, 53757 Sankt Augustin, Germany; 2 Enamine Ltd., Chervonotkatska Street 78, 02094 Kyiv, Ukraine; 3 Taras Shevchenko National University of Kyiv, Volodymyrska Street 60, 01601 Kyiv, Ukraine; 4 Bienta/Enamine Ltd., Chervonotkatska Street 78, 02094 Kyiv, Ukraine; 5 Chemspace, Ilukstes iela 38-5, LV-1082 Riga, Latvia

**Keywords:** chemical space, bromodomains, BRD4 inhibitors, new chemical entities

## Abstract

We introduce SAR by Space, a concept to drastically accelerate structure-activity relationship (SAR) elucidation by synthesizing neighboring compounds that originate from vast chemical spaces. The space navigation is accomplished within minutes on affordable standard computer hardware using a tree-based molecule descriptor and dynamic programming. Maximizing the synthetic accessibility of the results from the computer is achieved by applying a careful selection of building blocks in combination with suitably chosen reactions; a decade of in-house quality control shows that this is a crucial part in the process. The REAL Space is the largest chemical space of commercially available compounds, counting 11 billion molecules as of today. It was used to mine actives against bromodomain 4 (BRD4). Before synthesis, compounds were docked into the binding site using a scoring function, which incorporates intrinsic desolvation terms, thus avoiding time-consuming simulations. Five micromolar hits have been identified and verified within less than six weeks, including the measurement of IC_50_ values. We conclude that this procedure is a substantial time-saver, accelerating both ligand and structure-based approaches in hit generation and lead optimization stages.

## 1. Introduction

Over the last decades, medicinal chemistry has made a major breakthrough into expanding the accessible chemical space, which is estimated to amount to approximately 10^63^ possibilities [1,2]. With introducing high-throughput technologies in various areas of drug discovery, as well as major advancements in computational techniques, it became possible to mine the “observable” chemical space in an efficient manner [2,3]. A number of papers have appeared describing efforts to enumerate as many as possible compounds which might be relevant to drug discovery, the “Generated DataBase” (GDB) by Reymond group being one of the most prominent examples of such studies [4]. Combined with the current highly improved virtual screening capabilities, such “virtual” databases are a promising source of novel lead structures for various biological targets [2]. The major drawback of virtually generated databases is related to synthetic accessibility of the virtual hits obtained after in silico screening. One of the possible ways to address this problem relies on the use of commercially available off-the-shelf compounds (e.g., ZINC database [5,6]); in this case, however, patentability issues might arise when the initial hit is progressed further. Several big pharma companies, as well as academic institutions, reported the construction of large virtual libraries based on in-house validated or reported reactions (e.g., Merck’s MASSIV space [7] or Pfizer’s PGVL [8]) and internally or commercially available reagents [9,10], respectively [2]. Over a decade ago, we used a similar idea to generate the so-called REAL Database [11,12], which is covered by compounds easily synthesizable from the available collection of pre-validated building blocks through a set of validated reactions, and deliverable with high feasibility within short times upon demand.

Traditionally, in silico screening was mostly conducted in the so-called “enumerated” libraries that range in size from a few thousand to, recently, a few hundred million molecules (7 × 10^8^, the REAL Database) [13]. Being inaccessible to any enumeration-based method, much larger numbers than the traditional libraries can only be reached when exploiting chemical spaces that are created using combinatorics [3,14]. With a focus on commercial availability, we have used this idea to create a combinatorial reaction-driven chemical space, the REAL *Space* (version 1: 6.5 × 10^8^, currently: 1.1 × 10^11^ molecules). The molecules in the REAL *Space* are not stored as the final enumerated structures, but as the building blocks and the chemical reactions of how to combine them to obtain the final products. This is not to be confused with the well-known, enumerated REAL *Database* (previous version used in [15]: 1.7 × 10^8^; currently: 7 × 10^8^) [12], that is magnitudes smaller than the REAL Space. Both REAL *Database* and REAL *Space* are continuously being further expanded; the REAL Space counts over 11 billion tangible compounds today. Navigation through such large combinatorial spaces can be accomplished with software that builds up the results on-the-fly, assembling the hits during the search process [16]. The underlying algorithm, Feature Trees (FTrees), describes molecules as trees, the nodes of which contain physicochemical information about the contributing atoms. A similarity value between two virtual molecules is computed by creating the best possible alignment of two trees (cp. below for more details). It should be noted that due to the huge size of the REAL Space, as well as the fact that the structures of the final compounds are not enumerated and retrieved prior to the search, classical substructure-based searches become very complicated and time-consuming as compared to the similarity-based one.

**Figure 1:**
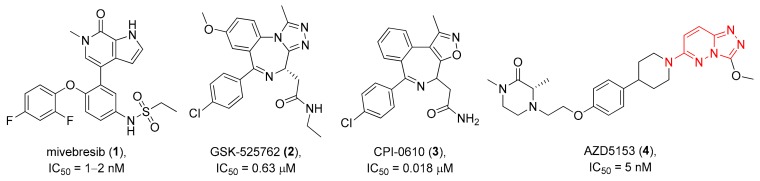
Investigational drugs—bromodomain 4 (BRD4) inhibitors.

In a recent paper, we demonstrated the utility of the REAL Database for the straightforward fragment-based discovery of novel bromodomain-containing protein 4 (BRD4) inhibitors [15]. This extensively studied member of the bromo- and extra terminal domain (BET) family has been related to numerous diseases including cancer, human immunodeficiency virus (HIV) infection, cardiovascular diseases, inflammation, and central nervous system (CNS) disorders [17,18,19,20,21,22]. Several BRD4 modulators have reached clinical trials as anti-cancer agents, e.g., Mivebresib (ABBV-075, **1**), GSK-525762 (**2**), CPI-0610 (**3**), or AZD5153 (**4**) [18,22,23,24] (Figure 1). Unfortunately, none of them has reached higher phases (i.e., at least phase 2) in clinical studies so far. The known molecules obviously fulfill pharmacophoric needs to bind to the target, but none of them seems to have the perfect structure to become a drug. Therefore, new bromodomain ligands with pharmacophoric features but of slightly different structure are of significant interest. For cases like this, fuzzy similarity descriptors capturing pharmacophore-like features such as the FTrees algorithm [25] are suitable as these compute similarities between molecules based on physico-chemical properties of parts of the molecule.

In our work mentioned above [15], the REAL Database was used as a source of highly feasible compounds for fragment evolution using the “SAR by catalog” approach. Although the proposed methodology was efficient and could deliver low-micromolar hits in a timely manner, it required synthesis and biological evaluation of thousands of analogs for the initially discovered active fragments. This approach becomes even less feasible when much larger REAL Space is considered as the compound source. In this work, we have developed an alternative strategy for hit expansion which was based on virtual screening for analogs in the REAL Space, followed by synthesis and biological evaluation of a small series of virtual hits thus identified.

## 2. Results and Discussion

**Figure 2:**
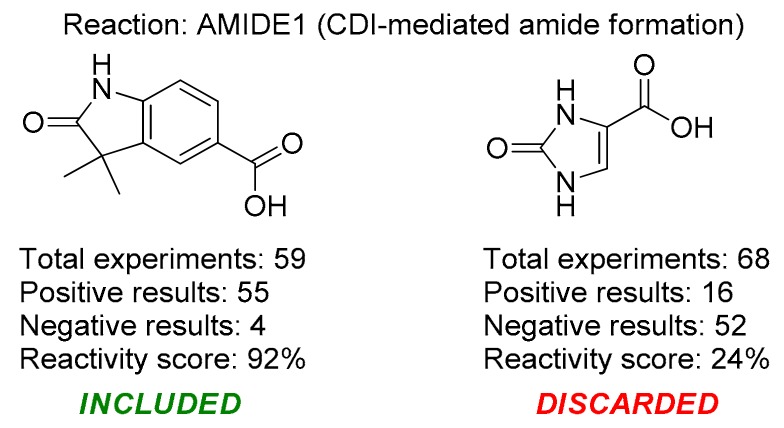
Selection of building blocks for REAL Space construction.

The construction of the first version of the REAL Space, which was used in this project, relied on 106 well-validated chemical transformations and ca. 130,000 off-the-shelf building blocks. Statistical data on the reactivity collected from over a decade was used to assign the reactivity score for each combination of building blocks and the corresponding reactions, and only the combinations with the highest scores were selected (the score assignment might also include a visual inspection by an expert) (Figure 2). This resulted in 54,548 well-validated building blocks, which were used to generate the REAL Space, version 1, containing 647,141,139 molecules [12].

The REAL Space navigation uses the query and fragments it in multiple ways (for further details, see the original FTrees-FS publication [16]). Starting with a query fragment, the chemical space fragments (that correspond to building blocks) are searched in such a way that the user-imposed target similarity is matched as good as possible. The results are built-up by adding one result fragment to the next one while continuously optimizing the similarity towards the given “target similarity” (cp. above). The similarity is defined through the best possible alignment of query fragments to REAL Space fragments, using the FTrees graphs as trees to be aligned [25].

**Figure 3:**
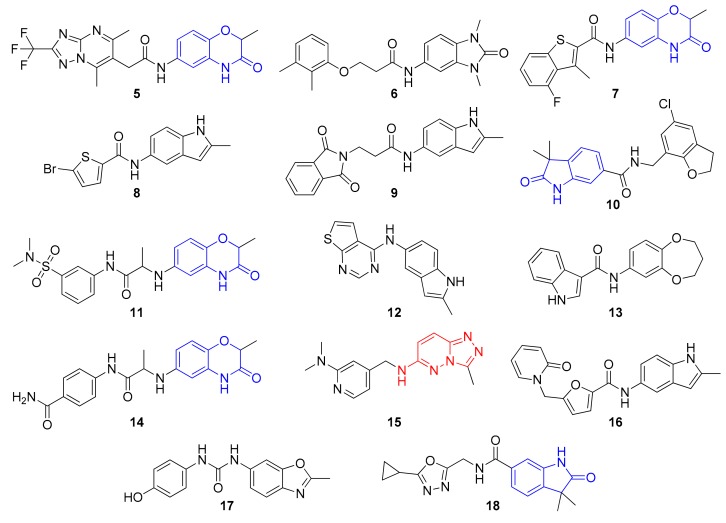
Fourteen compounds from the previous work [15] used as the queries to mine from the REAL Space.

The scope of this work was to increase the set of actives—as a second round after a first study—in a fast follow-up. During the first work [15] 14 compounds were found which showed high thermal shift and enzyme inhibition at 40 µM in a time-resolved fluorescence resonance energy transfer (TR-FRET) assay against BRD4 but no measurable IC_50_. To find close neighbors with measurable IC_50_s those 14 molecules (Figure 3) were used as queries to mine from the REAL Space (version 1) [12]. For each query molecule, 5000 similar molecules were retrieved from the REAL Space using the FTrees-FS algorithm (cp. below) [16]. The resulting molecules were filtered for duplicates. As for post-processing, the hit molecules were docked (PDB structure 3mxf) using the pharmacophoric constraint that the H-bond donor on Asn140 had to be met. Molecules for testing were picked in SeeSAR [26] with putting attention not only to a good predicted binding affinity but as well good torsion qualities [27], plausible interactions and good physicochemical parameters. A set of 32 compounds was selected for synthesis.

**Figure 4:**
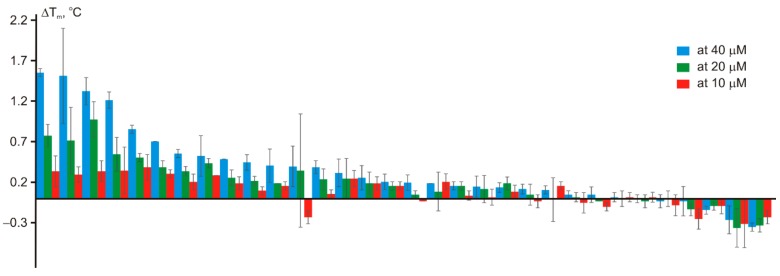
Thermal shift values (ΔT_m_) for the 32 synthesized compounds.

The compounds were synthesized according to the standard protocols for typical parallel synthesis transformations, including amide/sulfonamide coupling, reductive amination, urea synthesis, arylation/alkylation, etc. The synthesis was completed in a three-week period and all the 32 compounds could actually be synthesized. The products obtained were subjected to thermal shift assay (TSA) using recombinant, truncated, His-tagged bromodomain 1 of BRD4 [28] at 40, 20, and 10 μM (Figure 4). The hit selection criteria followed those described in our previous publication [15]:

|Δ*T_m_*| ≥ |Δ*T_m,av_*| + 3|σ(Δ*T_m_*)| (1)

|Δ*T_m_*| ≥ 2|σ(*T_m,DMSO_*)| (2)

where Δ*T_m_*—thermal shift caused by the compound; Δ*T_m,av_*—mean thermal shift value within the plate; σ(Δ*T_m_*)—standard deviation of the thermal shift values within the plate; σ(*T_m,DMSO_*)—standard deviation of the melting temperature values obtained for the control samples.

**Figure 5:**
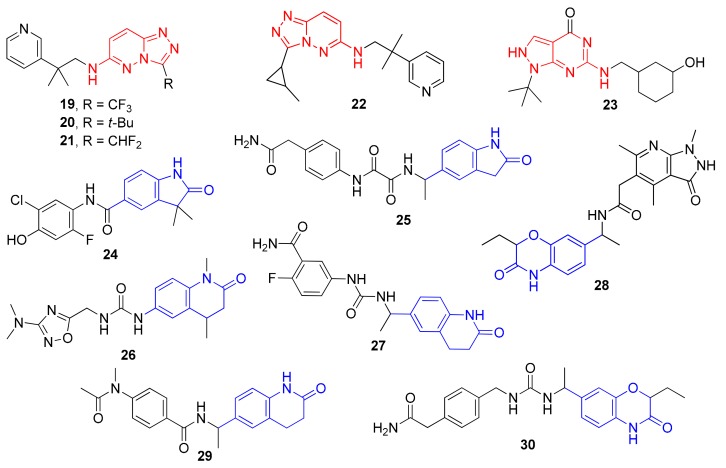
Two structural series identified after the thermal shift assay.

**Figure 6:**
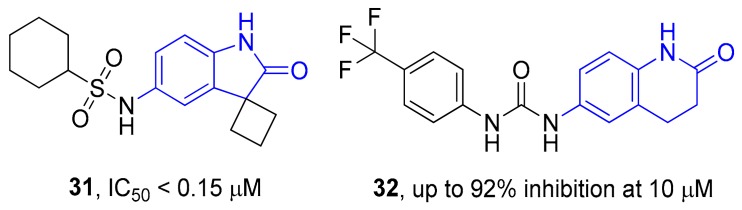
Known BRD4 inhibitors—analogs of the series identified in this work.

As a result, 12 hits **19–30** were identified which represented two distinct structural series (Figure 5). One of these series (6-amino [1,2,4]triazolo[4,3-*b*]pyridazine derivatives) has been well-documented since its representative AZD5153 (**4**) has recently entered Phase Ib clinical trials against hematologic malignancies [24]. The other one (5-substituted indolin-2-ones, 6-substituted 3,4-dihydroquino-lin-2(1*H*)-ones, and their analogs) is much less studied; only a few representatives with modest structural similarity can be found (Figure 6) [29,30].

**Figure 7:**
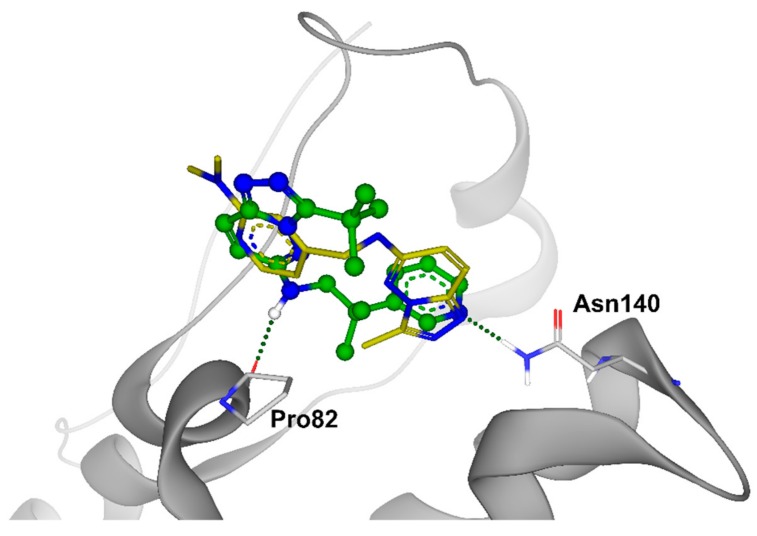
Binding poses of the molecules **15** (in yellow) and **20** (in green). The small changes of the molecular structure result in very different binding modes.

Dose-response curves were built for these compounds, and for 5 of them, IC_50_ values were determined in TR-FRET assay as an average from three independent experiments (Table 1). Comparing the resulting hit molecules with the corresponding initial queries, one can see considerable structural differences. Low Tanimoto similarity (calculated from Morgan fingerprints using RDKit in KNIME) indicates that those hits could be easily be missed using a classical similarity and even sub-structural search. The relatively high FTrees similarity explains why they were not missed in the REAL Space search: The molecules still have very similar pharmacophoric properties in a similar arrangement. As the FTrees algorithm is fuzzy regarding the connectivity this can lead as well to significantly different binding modes of query and hit. This is exemplary shown in Figure 7 for compound **20** and its respective query molecule **15**. The shift of the nitrogen atom to the β position of the pyridine ring, as well as the different linker length, led to a completely flipped binding mode. It is also important to stress that the starting points of this study which led to the hits (compounds **19**–**22**) had no significant activity against BRD4 in the functional (TR-FRET) assay; they were identified only in the binding assay (TSA).

## 3. Materials and Methods

### 3.1. General

All tested compounds were obtained from Enamine Ltd. (Kyiv, Ukraine). Stock solutions of the tested compounds were prepared in 100% DMSO and were stored at −20 °C until use. The bromodomain 1 of BRD4 was expressed using pNIC28-Bsa4 plasmid vector with an insert representing domain 1 of BRD4 (44–168 AA, sequence entry O60885.1 in UniProtKB Database [31]), *N*-terminus His_6_-tag and 16-amino acid linker.

### 3.2. Thermal Shift Assays

All thermal shift assay (TSA) experiments with BRD4 protein were performed using ViiA™7 real-time PCR System equipped with 384-well heat block (Applied Biosystems, Waltham, MA, USA). General TSA methodology was adopted from the literature [32,33,34] and experimentally modified in order to optimize conditions for measuring BRD4 melting temperature shifts upon interaction with small molecules. The optimal buffer composition for the TSA procedure was determined as described previously. [15] Buffer consisting of 50 mM Phosphate-Na, 100 mM NaCl, pH = 7.5 was selected for BRD4 screening in this study. Purified BRD4 protein was pre-mixed with SYPRO Orange dye (Thermo Fischer Scientific, Cat. S6650, 5000x stock, Waltham, MA, USA) to prepare a master mix at 4 μM protein and 6× dye concentrations. Tested compounds were added to the protein-dye master mix at 40, 20, or 10 μM at 1% final DMSO concentration and incubated at 4 °C for 1 h in MicroAmp^®^ optical 384-well reaction plates (ThermoFisher, Cat. 4309849, Waltham, MA, USA) sealed with optical sealing film (ThermalSeal RT2, Excel Scientific, Cat. TS-RT2, Victorville, CA, USA). The volumes of all reaction mixtures were 10 µL (4 µg BRD4 per well). The reaction plates were then kept at ambient temperature (22–24 °C) for an additional 15 min to ensure protein-compound interactions. Thermal scanning was performed by raising the temperature to 40 °C at 1.6 °C/min without signal detection followed by 40 °C to 90 °C temperature ramp at 0.05 °C/s with constant fluorescence intensity reading at 1-sec intervals using EX470/EM623 nm filter set.

Primary screening of the whole test set of 49 compounds was carried out in singletons. The raw data of dye fluorescence intensity change upon protein melt were exported using the ViiA7 RUO software (Applied Biosystems/Thermo Fischer Scientific). Further data visualization, curve fitting, melting temperature calculations on the raw fluorescence data were performed using custom-made Microsoft Excel scripts. The peak of the first derivative for the fluorescence curve was used to define melting temperature (*T_m_*). Averaged *T_m_* values for the control wells containing only the protein, dye and 1% DMSO were used as a reference point to determine melting temperature shifts (Δ*T_m_*).

### 3.3. TR-FRET Assays and IC_50_ Measurements

All TR-FRET assays and IC_50_ measurements were performed using BRD4 (BD1+BD2) TR-FRET assay kit from BPS Bioscience (www.bpsbioscience.com) following the standard procedure [35].

### 3.4. Molecular Docking Studies

The 14 best molecules—regarding their thermal shift—from a previous publication [15] were used as queries to mine from the REAL Space (version 1, containing 647 million molecules) [12]. For each query molecule, 5000 similar molecules were retrieved from the REAL Space using the FTrees-FS algorithm [16]. The resulting molecules were filtered for duplicates. All molecular batch processing was accomplished using the KNIME package v3.5.3 [36], containing BioSolveIT nodes as well as all community nodes. All molecular initialization and consistency was ensured through NAOMI and ProToss functionalities [37,38]. As post-processing step, the hit molecules were docked. To ensure a reasonable docking, six different crystal structures of BRD4 were loaded from the PDB (codes: 3mxf, 3zyu, 4e96, 4nud, 5igk, 5uvw) and superposed using SeeSAR v7.2 [26]. The binding modes of all co-crystalized ligands and their key-interaction points inside the binding pockets were analyzed. Those were found to be mainly H-bonds to Asn140, Asp88, and water-mediated H-bonds to Ile144 and Gln85. Re-docking of known binders from the crystal structures showed best results with the crystal structure 3mxf. The active site was defined as a 7.5 Å around the ligand JQ1. To improve the docking results a pharmacophore was applied, which was that the H-bond donor on Asn140 had to be met. This had shown the best ranking of known binders in the test-dockings trying different pharmacophores. All 64,683 molecules were docked with 5 poses per molecule using FlexX [39] and subsequently scored using HYDE [40,41]. Thirty-three molecules for testing were picked in SeeSAR [26].

## 4. Conclusions

**Figure 8:**
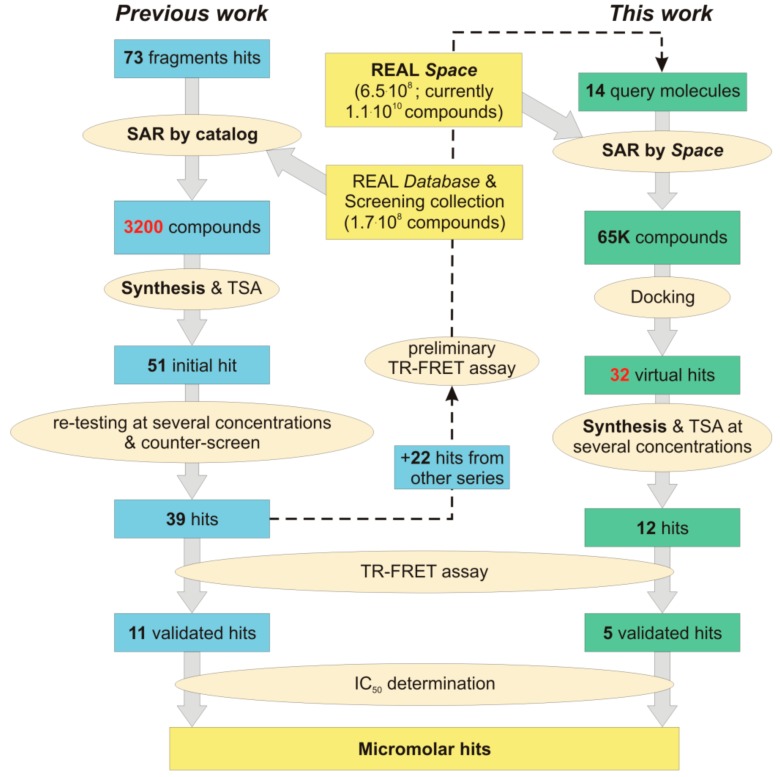
The workflow of the project described in this work compared to the fragment hit expansion used in our previous publication [15].

This work demonstrates a much-accelerated structure-activity relationship (SAR) exploration via fast navigation through large chemical space. Using query compounds to mine in the reaction-driven REAL Space we identified molecules with similar pharmacophores but different Bemis–Murcko scaffolds. As expected, the hits show low Tanimoto similarity as compared to the original query structures. Pocket compatibility was rapidly assessed using parallel docking. Compared to both the classical as well as fragment-based hit expansion approaches (in particular the one used in our previous work [15]), the methodology proposed herein enabled much faster evaluation of SAR hypotheses (Figure 8). More importantly, the strategy required much fewer (100-fold) compounds to be synthesized and subjected to “wet” screening, which resulted in higher cost-efficiency. Compared to previous works, the chemical space that served as a pool for the compound selection to mine in is orders of magnitude larger. Notably, all the selected compounds could be synthesized within a 3-week timeframe. In summary, the approach described here has the potential to significantly streamline the early drug discovery process in general.

## Figures and Tables

**Table 1:** Most active BRD4 inhibitors identified after TR-FRET (time-resolved fluorescence energy transfer) assay.

#	Query in the REAL Space	Hit	Similarity		Δ*T_m_* (°C) at			IC_50_ (μM)
			Tanimoto	FTrees	40 μM	20 μM	10 μM	
1	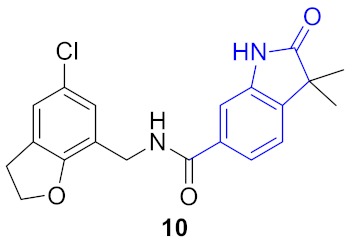	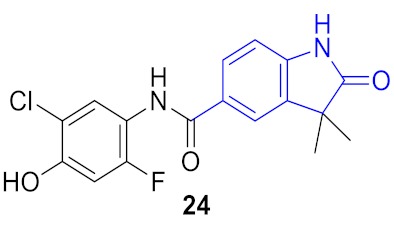	0.456	0.956	0.71 ± 0.01	0.39 ± 0.08	0.31 ± 0.05	10.7
2	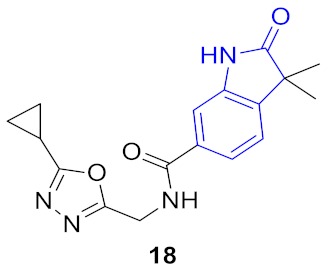	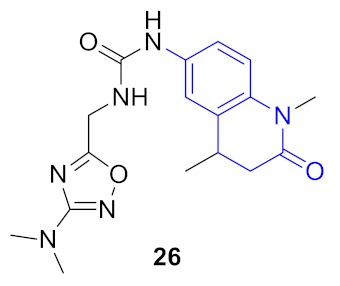	0.277	0.920	1.52 ± 0.59	0.72 ± 0.41	0.30 ± 0.10	26.4
3	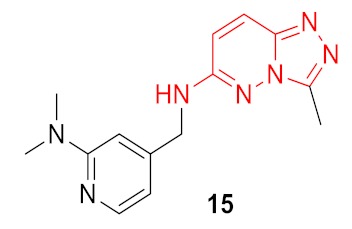	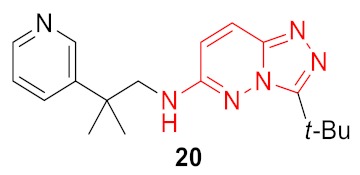	0.323	0.933	1.33 ± 0.17	0.98 ± 0.22	0.34 ± 0.13	44.6
4		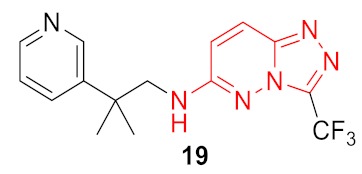	0.333	0.953	1.56 ± 0.05	0.78 ± 0.14	0.34 ± 0.19	68.2
5	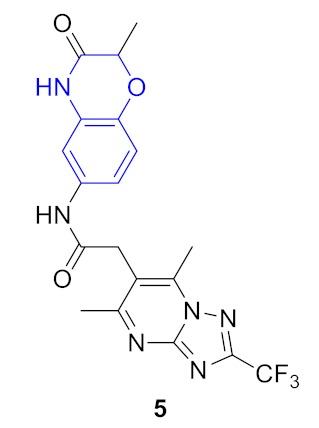	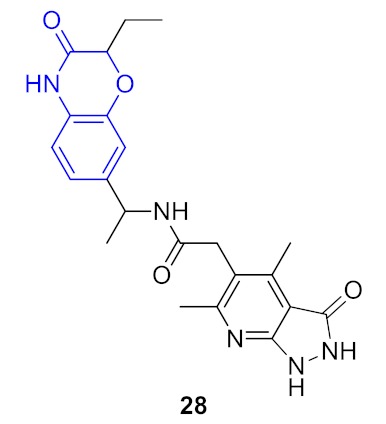	0.356	0.932	0.53 ± 0.25	0.44 ± 0.06	0.29 ± 0.01	141
